# Integration of mRNA and microRNA analysis reveals the molecular mechanisms underlying drought stress tolerance in maize (*Zea mays* L.)

**DOI:** 10.3389/fpls.2022.932667

**Published:** 2022-09-29

**Authors:** Peng Jiao, Ruiqi Ma, Chunlai Wang, Nannan Chen, Siyan Liu, Jing Qu, Shuyan Guan, Yiyong Ma

**Affiliations:** ^1^ College of Life Sciences, Jilin Agricultural University, Changchun, China; ^2^ Joint International Research Laboratory of Modern Agricultural Technology, Ministry of Education, Jilin Agricultural University, Changchun, China; ^3^ College of Plant Science, Jilin University, Changchun, China

**Keywords:** drought stress, maize, microRNA, mRNA, miR408a

## Abstract

Drought is among the most serious environmental issue globally, and seriously affects the development, growth, and yield of crops. Maize (*Zea mays* L.), an important crop and industrial raw material, is planted on a large scale worldwide and drought can lead to large-scale reductions in maize corn production; however, few studies have focused on the maize root system mechanisms underlying drought resistance. In this study, miRNA–mRNA analysis was performed to deeply analyze the molecular mechanisms involved in drought response in the maize root system under drought stress. Furthermore, preliminary investigation of the biological function of miR408a in the maize root system was also conducted. The morphological, physiological, and transcriptomic changes in the maize variety “M8186” at the seedling stage under 12% PEG 6000 drought treatment (0, 7, and 24 h) were analyzed. With prolonged drought stress, seedlings gradually withered, the root system grew significantly, and abscisic acid, brassinolide, lignin, glutathione, and trehalose content in the root system gradually increased. Furthermore, peroxidase activity increased, while gibberellic acid and jasmonic acid gradually decreased. Moreover, 32 differentially expressed miRNAs (DEMIRs), namely, 25 known miRNAs and 7 new miRNAs, and 3,765 differentially expressed mRNAs (DEMRs), were identified in maize root under drought stress by miRNA-seq and mRNA-seq analysis, respectively. Through combined miRNA–mRNA analysis, 16 miRNA–target gene pairs, comprising 9 DEMIRs and 15 DEMRs, were obtained. In addition, four metabolic pathways, namely, “plant hormone signal transduction”, “phenylpropane biosynthesis”, “glutathione metabolism”, and “starch and sucrose metabolism”, were predicted to have important roles in the response of the maize root system to drought. MiRNA and mRNA expression results were verified by real-time quantitative PCR. Finally, miR408a was selected for functional analysis and demonstrated to be a negative regulator of drought response, mainly through regulation of reactive oxygen species accumulation in the maize root system. This study helps to elaborate the regulatory response mechanisms of the maize root system under drought stress and predicts the biological functions of candidate miRNAs and mRNAs, providing strategies for subsequent mining for, and biological breeding to select for, drought-responsive genes in the maize root system.

## Introduction

Maize is the largest food crop worldwide (Koester et al., 2021); therefore, ensuring stable maize production is of considerable significance to the sustainable development of agricultural output. As a stress-sensitive plant, maize is susceptible to interference and damage from stress conditions. With the development of modern industries, environmental challenges, such as reduction of arable land, deterioration of soil quality, shortage of freshwater resources, global warming, and frequent natural disasters, have become increasingly serious. Maize yields are limited by various abiotic stressors, including drought stress, which is an important limiting factor for crop yield that seriously threatens the sustainable development of global agriculture. Therefore, research on drought resistance of maize is essential.

Drought stress-induced signals undergo a series of transmission processes to finally regulate the expression of specific genes, leading to drought resistance at the tissue, cell, and physiological/biochemical levels in plants. The products encoded by these specific genes can generally be divided into two categories. First, functional proteins, such as osmolytes, various proteases, aquaporins, molecular chaperones, and LEA protein enzymes, related to active oxygen scavenging, among others, are directly involved in various biochemical environmental stress response resistance processes, resulting in drought tolerance effects (Battaglia et al., 2008; Ramanjulu and Bartels, 2022). Second, regulatory proteins act upstream of genes encoding functional proteins and mediate related signaling, for example, transcription factors and various kinases involved in signaling cascades (Tõldsepp et al., 2018; Su et al., 2019). At the molecular level, plant drought stress transcriptional regulatory networks comprise two different regulatory pathways: abscisic acid (ABA)-dependent and ABA-independent (Bulgakov et al., 2019).

Recently, increasing studies have shown that numerous miRNAs are involved in plant stress response processes; miRNAs are non-coding single-stranded RNA molecules of 16–29 nucleotides, encoded by endogenous genes, which have characteristic hairpin structures, and function at the post-transcriptional level. Degradation of target mRNA or translation inhibition by miRNAs to regulate gene expression is crucial for control of gene expression in eukaryotic cells (Cui et al., 2017; Pegler et al., 2019). Many plant miRNA genes map to intergenic or intronic genome regions (Voinnet, 2009), and some form clusters, which can be co-transcribed to form a common primary miRNA (pri-miRNA). miRNA was first discovered in nematodes (Lee et al., 1993). Subsequently, numerous miRNAs were also predicted in rice (Jian et al., 2010), Arabidopsis (Sunkar and Zhu, 2004), maize (Kaur et al., 2020), and other plants, and many have been successfully experimentally verified (Fang et al., 2014; Luan et al., 2015; Kravchik et al., 2019).

Many studies have shown that miRNAs regulate plant growth and development not only under normal conditions but also under abiotic stress conditions such as drought (Arshad et al., 2017; Zhang et al., 2018), saline-alkali (Zhai et al., 2014; Zhang et al., 2020), and low temperature (Yang et al., 2016; Zhou and Tang, 2019). Plants are also regulated to enhance tolerance to stress. Under stress, inducing or inhibiting the expression of the specific genes at the post-transcriptional level caused by different miRNA expression levels leads to the accumulation of individual substances or changes in metabolic pathways. It ultimately helps the plant make adaptive adjustments to improve plant stress resistance.

To date, numerous small-molecule RNAs have been identified in multiple organisms using various technologies, and this process has been accelerated by the emergence of high-throughput small-molecule sequencing methods and bioinformatics technologies. Although regulation of target gene expression generates robust regulatory networks, understanding this represents only an initial research step. Such networks ensure the operation of various physiological processes, and miRNAs are essential regulators of plant drought response (Akdogan et al., 2016). The first miRNAs identified as related to drought in rice were those of the miR169 family, which includes 17 members with 9 different mature sequences. miR169 is more sensitive to drought stress in roots than in buds (Zhao and Srivastava, 2007). Furthermore, miR169a and miR169c of Arabidopsis and miR169 of tomato are inhibited by drought (Ni et al., 2013). In transgenic tomatoes that overexpress miR169c, target gene expression is downregulated, and transgenic plants have lower transpiration rates and reduced stomatal openness relative to wild-type (WT) controls; hence, the reasons underlying miR169c involvement in tomato drought stress responses have been determined (Zhang et al., 2011). MiR408 family is a well-recognized class of conserved miRNAs in plants, which mainly responds to adversity stress. Under drought stress, the expression of miR408 in alfalfa, barley, chickpea, and poplar also increases, while the expression of miR408 in rice and Arabidopsis is inhibited by drought stress (Ma et al., 2015; Hang et al., 2021). Zhang et al. (2017) found that miRNA408-mediated target genes can affect copper ion homeostasis in chloroplast, which, in turn, results in enhancement of stress resistance, photosynthetic rate, and yield of rice; Ma et al. (2015) proved that miR408 in Arabidopsis can mediate target genes to improve stress resistance and oxidative stress response in plants under the effects of low temperature, drought, oxidation, and osmotic pressure. Therefore, miRNAs are important in the response of plants to drought stress.

In this study, we used 12% PEG 6000 to conduct experiments simulating hydroponic drought treatment using three-leaf stage maize plants of the inbred line “H8186”, with a treatment duration of 0, 7, and 24 h. Observation of the maize root system phenotype before and after drought treatment was conducted and physiological indices were measured. To investigate the molecular mechanism involved in drought stress responses, nine small RNA (sRNA) sequencing libraries and nine mRNA libraries were constructed, with three biological replicates each, and miRNA-seq and mRNA-seq data were generated and analyzed. Our findings contribute to understanding of post-transcriptional regulation of the maize root system under drought stress, and provide insight into drought tolerance in the grass family. Furthermore, high-throughput sequencing and bioinformatic analyses were adopted to identify candidate miRNAs and mRNAs involved in maize root responses to drought stress, providing new strategies for creation of novel drought-tolerant maize germplasm and cultivation of innovative varieties.

## Materials and methods

### Plant materials and experimental design

The plant material used in this experiment was the maize (Zea Mays L.) inbred line, M8186. All plant materials were preserved by Jilin Agricultural University Jilin Key Laboratory of Crop Molecular Breeding. Maize was grown in the greenhouse of the experimental base of Jilin Agricultural University at 27°C ± 2°C, with a 14-h light/10-h dark cycle. Three-leaf stage maize plants were randomly divided into drought (7 and 24 h) and control groups. Drought stress was simulated by watering plants with 12% PEG 6000; plants in the control group were watered as usual throughout the experiment. Morphological observation of maize plants was performed and experimental samples were collected before (0 h) and after (7 and 24 h) drought stress. Collected maize root system samples were frozen in liquid nitrogen for 30 min, then quickly transferred to an ultra-low-temperature refrigerator at –80°C. Three biological replicates were used for each time point.

Maize inbred lines used for genetic transformation included the drought-tolerant inbred line, GSH9901, which was obtained by Prof. Guan Shuyan’s research group at the Joint International Research Laboratory of Modern Agricultural Technology (43° 47′ 56′ N, 125° 24′ 2′ E), Ministry of Education, after many years of field screening at multiple locations. The materials used were high-generation stable maize inbred lines, which grew in a greenhouse in well-mixed soil (1:1 ratio, forest soil:vermiculite) at 28°C/26°C, with a 12-h light/12-h dark cycle. To analyze changes in physiological indices and expression levels of ROS-related marker genes under drought, 12% (w/v) polyethylene glycol 6000 (PEG 6000) was used to simulate drought by treating the following groups of three-leaf stage maize plants: GSH9901 (WT control), miR408a-OE (maize lines generated using a T-DNA insertion system to overexpress miR408a: miR408a-OE#3 and miR408a-OE#9), and miR408a-KO (knockout maize mutants generated using the CRISPR/Cas9 system: miR408a-KO#2 and miR408a-KO#7). Plants were grown in soil under the conditions described above. All experiments were repeated three times, and >10 plants were used for each measurement.

### Measurement of physiological indices

Physiological indices were measured using frozen maize root samples. High-performance liquid chromatography was applied to determine jasmonic acid (JA), ABA, gibberellic acid (GA3), and brassinolide (BR) content in the maize root system (Pan et al., 2010). Peroxidase activity was measured using the guaiacol method (Jiao et al., 2022b), and lignin content was determined in strict accordance with the operation process specified for testing kits from Suzhou Michy Biomedical Technology Co., Ltd. Reduced glutathione (GSH) and trehalose content were determined by UV spectrophotometry (Yang et al., 2021a; Kang et al., 2021).

### sRNA isolation, library construction, and high-throughput sequencing

Root samples of maize were collected at 0, 7, and 24 h under drought treatment, respectively. Three biological replicates were prepared for each sample, and each biological replicate was collected from eight plants. Samples were quickly frozen in liquid nitrogen, and total RNA was extracted and then sequenced. Three groups of RNA samples were prepared (WT: roots with watering, DSSD: roots with drought stress 7 h, and DSFD: roots with drought stress 24 h), each group had three biological replicates, and each group of samples was mixed and combined with RNA. Therefore, a total of nine samples were used to build an sRNA library. The construction of the sRNA library and high-throughput sequencing was completed by Shanghai Majorbio Bio-pharm Technology Co, Ltd. MiRNA and mRNA data were uploaded to the National Center for Biotechnology Information (PRJNA796152 and PRJNA793522) on 31 December 2021.

### Identification of known and novel miRNAs

To identify known and novel miRNAs, bowtie software was used to align mapped sRNAs with a specified range of sequences in miRBase. Then, miRNA sequences were compared with the Rfam database, and rRNA, tRNA, snRNA, and snoRNA sequences were removed. MISA software was used to remove repeat sequences. PlantNATsDB analysis was used to remove sequences from the plant NAT-siRNATAS database, and UEA sRNA tools software was used to remove plant ta-siRNA sequences. After removal of interfering sRNA sequences, the unique hairpin structures of miRNA precursors were used to predict new miRNAs from remaining sRNAs.

### Identification of known and novel mRNAs

After acquisition of read count data of genes/transcripts, the differential expression analysis of genes/transcripts between samples was performed for multi-sample (≥2) projects to identify the differentially expressed genes/transcripts between samples and in turn investigate the function of differentially expressed genes/transcripts. In this module, DESeq2 software was used to conduct statistical analysis of differentially expressed genes/transcripts, and the detailed table of differentially expressed genes/transcripts (expression of differentially expressed genes/transcripts in a single comparison group) and the statistical table of differentially expressed genes/transcripts (expression of differentially expressed genes/transcripts in multiple comparison groups) were included.

### Prediction of miRNA targets and association analysis

The psRobot (http://omicslab.genetics.ac.cn/psRobot/index.php) software was used to predict the target genes of mature miRNAs. psRobot is a plant sRNA analysis tool and is divided into an online version and a local version. The local version has the function of reference sequence alignment, prediction of precursor and mature miRNAs, prediction of sRNA target genes, and degradation group analysis. Mature miRNA sequences were responsive to drought, identified in maize roots, and were used as custom miRNA sequences. The parameters for target prediction were set by default. The miRNAs and their target genes were counted, and a miRNA–mRNA network was constructed by Cytoscape.

### Functional analysis of differentially expressed genes

Based on the principle of hypergeometric distribution, functional analysis of all genes with significantly altered expression in response to drought stress was conducted using the GO (http://www.geneontology.or) and Kyoto Encyclopedia of Genes and Genomes (KEGG; http://www.genome.jp/kegg/) databases. GO and KEGG enrichment analyses were performed using the OmicShare tool, a free online platform for data analysis (https://www.omicshare.com/tools).

### Expression validation of miRNA and their targets

Chen et al. (2005) developed a stem-loop primer real-time PCR method that could detect the expression level of mature miRNA. This method was the first to design a specific stem-loop RT primer for mature miRNA. The primer had a stem-loop structure, and about six bases at its 3’ end were reversely complementary to the 3’ end of miRNA, thereby specifically reverse transcription of miRNA. Then, miRNA was amplified by specific forward primers and universal reverse primers. The transcription of miRNA was reversed using the Taq Man micro RNA Reverse Transcription Kit. Then, two-step PCR was used to perform PCR amplification. The procedures were denaturation at 95°C for 30 s, denaturation at 95°C for 5 s, and extension at 60°C for 31 s, a total of 40 cycles. Three biological and three experimental replicates were performed for each sample.

Primer 5.0 software was used to design primers for miRNA target gene quantitative PCR. The main design principles were as follows: (1) The amplified length of the fragment was generally between 100 and 200 bp. (2) The primer did not contain a secondary structure, and the primers were between the primers. Dimers could not be formed. (3) The primer length was generally about 19–25 nt, and G or C should be avoided at the 3’ end. (4) The Tm value of the primer was generally about 60°C, and the GC content was about 40%–60%. All primers were synthesized by Kumei Biotechnology Co., Ltd. of Jilin Province. Target genes were reverse transcribed using the First-Strand c DNA Synthesis Kit. The PCR reaction was performed as follows: pre-denaturation at 94°C for 10 min, 94°C for 15 s, and 60°C for 1 min, for a total of 40 cycles. After the reaction, the dissolution curve was analyzed using the 2–ΔΔCT [ΔCT = CTtarget gene – CTinternal reference gene. ΔΔCT = ΔCTtreated – ΔCTcontrol] method (Livak and Schmittgen, 2001). Three biological experiment replicates were set up per gene. Details regarding the primers used for this assay are listed in **Supplementary Table S3**.

### β-Glucuronidase assay

β-Glucuronidase (GUS) histochemical staining of root systems from the miR408a transgenic maize inbred line, GSH9901, was performed; root systems from three-leaf stage plants were stained under vacuum in staining solution containing X-gluc (50×) and GUS staining PBS buffer. The specific steps for determining GUS activity were as described previously (Jiao et al., 2022a).

### Nitrotetrazolium blue chloride staining and oxidative stress analyses

Drought simulation experiments were carried out using three-leaf stage miR408a OE, miR408a KO, and WT plants, by treatment with 12% PEG 6000. Seedling leaves (the middle part of the second leaf in the three-leaf stage) were stained using nitrotetrazolium blue chloride (NBT), and the accumulation of superoxide anion (O_2_^−^) was quantitatively detected. Superoxide dismutase (SOD), catalase (CAT), and ascorbic acid peroxidase (APX) activities were determined by spectrophotometry. Malondialdehyde (MDA) content, proline content, relative water content, hydrogen peroxide concentration, and O_2_^−^ content were determined following the criteria described by Jiao et al. (2022a).

### Statistical analysis

All of the data were tested by analysis of variance using SPSS 19.0 software. The data are the mean ± standard deviation (SD) of three biological replicates. The significance was analyzed in the Student’s t-tests. The * and ** represent p < 0.05 and p < 0.01, respectively. The figures were prepared with GraphPad Prism 8.0.

## Results

### Effects of PEG-induced water stress on the morphological and biological characteristics of maize

**Figure 1:**
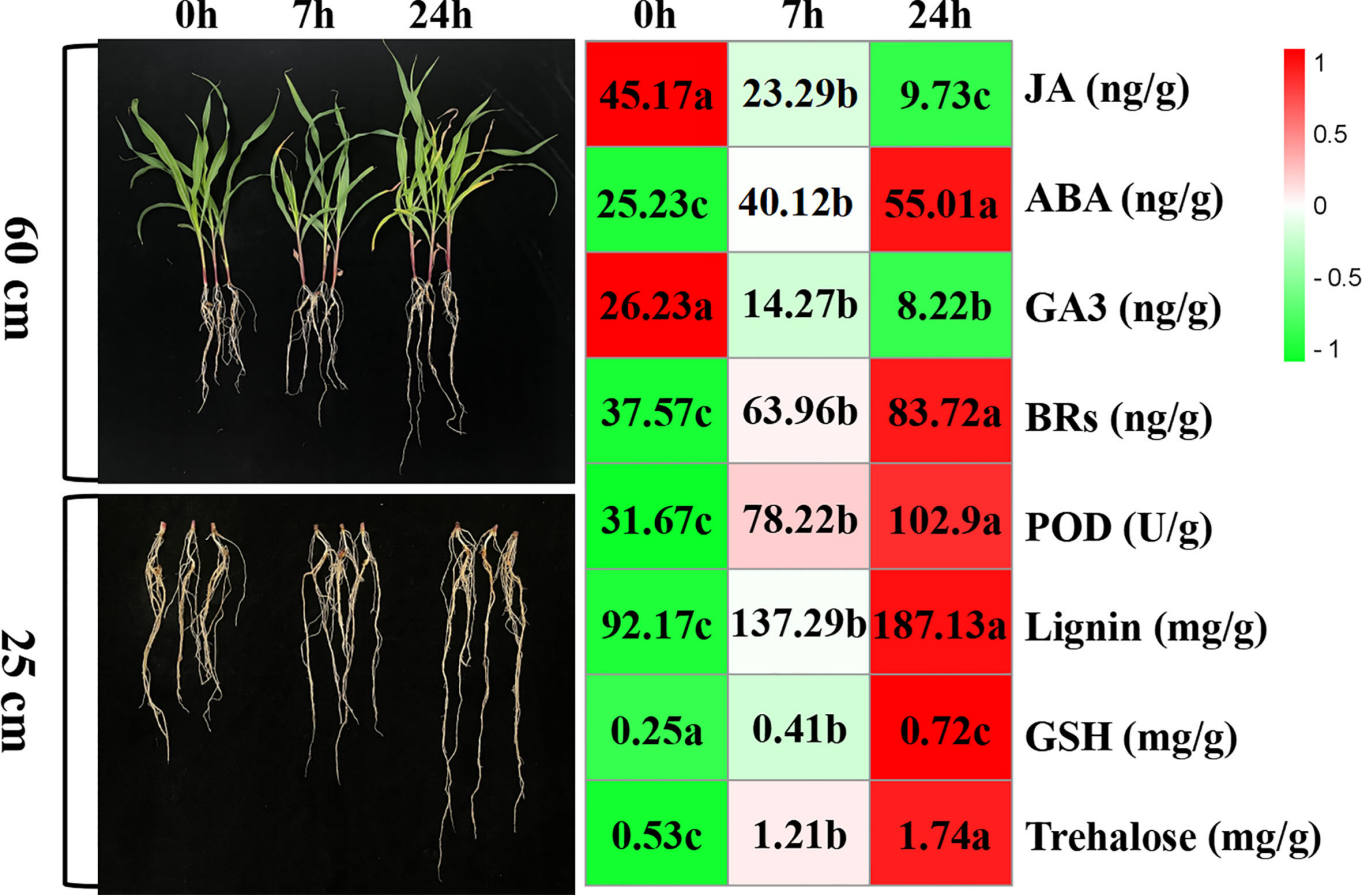
The morphology and physiochemical changes of maize variety “M8186” under drought stress. Values followed by different lowercase letters represent p ≤ 0.05.

The morphology of maize seedlings changed with increased duration of drought stress exposure. Compared with seedlings before drought stress, those exposed to drought stress for 7 h began to wither slightly, and the root length grew. Furthermore, after 24-h drought stress, seedlings had withered significantly and their root systems grew significantly. With prolonged drought stress, hormone levels, lignin content, and peroxidase activity in the root system were significantly affected; ABA, BRs, lignin, GSH, and trehalose content increased gradually, while peroxidase activity increased sharply, and GA3 and JA content decreased gradually (**Figure 1**).

### miRNA and mRNA sequencing data quality assessment

To elucidate the molecular mechanisms underlying maize root system responses to drought stress, nine root samples, including three biological replicates from control and drought stress-exposed plants, were additionally selected and subjected to mRNA and sRNA library sequencing using the Illumina HiSeq™ 2500 platform. Approximately 12.979–17.682 × 106 clean reads and 358.219–476.732 × 106 clean bases were obtained by miRNA sequencing, and useful reads (9.600–13.970 × 106) were screened according to sRNA length 18–32 nt. CG content ranged from 51.15% to 52.64%. Numbers of known and new miRNAs identified in each sample are presented in **Table 1**.

**Table 1:** Summary of miRNA sequencing datasets.

Sample	Clean reads(×106)	Clean bases(×106)	GC content (%)	Useful reads (18 nt–32 nt)(×106)	Known miRNA	Novel miRNA
WT	17.682	476.732	51.15	13.970	240	449
DSSD	15.499	431.279	52.64	11.525	231	188
DSFD	12.979	358.219	52.19	9.600	229	175

A total of 64.42 GB clean data were obtained by mRNA sequencing, representing > 6.57 GB per sample and 82.90–95.64 million clean reads for each of the four samples. Q30 base call rates were all > 93.63%. Hisat2 was used to map clean reads to the maize reference genome, with proportions of clean reads that could be mapped to the genome ranging from 88.47% to 89.77%. Uniquely mapped clean reads (85.07%–86.68%) were applied for subsequent analyses (**Table 2**).

**Table 2:** Summary of mRNA sequencing datasets.

Sample	Clean reads (×106)	Clean bases (×109)	Q30 (%)	Total mapped	Uniquely mapped
WT	47.253	7.05	94.35	88.47%	85.07%
DSSD	47.237	7.04	94.08	89.60%	86.68%
DSFD	49.672	7.39	94.46	89.77%	86.68%

### Analysis of differentially expressed miRNAs and differentially expressed mRNAs

**Figure 2:**
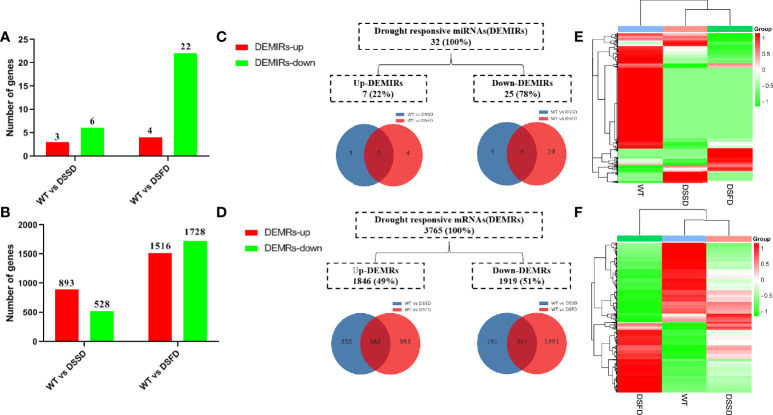
The expression profile of drought stress-regulated DEMIRs and DEMRs in maize roots. **(A, B)** Column diagram representing the numbers of DEMIRs and DEMRs. **(C, D)** Venn diagrams representing the numbers of DEMIRs and DEMRs, and the overlaps of sets obtained across two comparisons. **(E, F)** Heat map of all miRNAs and all mRNAs expression profiles before and after drought stress.

Numbers of both differentially expressed miRNAs (DEMIRs) and differentially expressed mRNAs (DEMRs) gradually increased with prolonged drought stress (**Figures 2A, B**). A total of 32 DEMIRS were identified, comprising 22% and 78% that were up- and downregulated, respectively. Furthermore, 3,765 DEMRs were identified, of which 49% and 51% were up- and downregulated, respectively (**Figures 2C, D**). A cluster heatmap of all miRNAs and mRNAs illustrated the significant differences in miRNA and mRNA expression levels in response to drought stress (**Figures 2E, F**).

### Functional analysis of DEMRs

**Figure 3:**
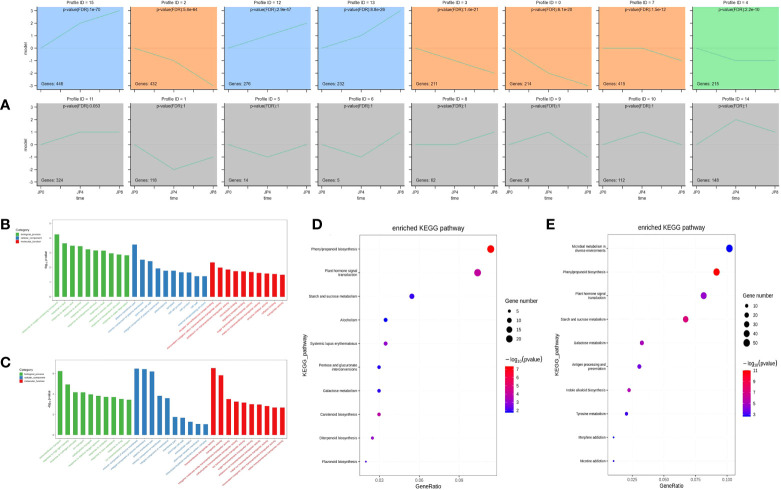
GO and KEGG enrichment analysis across three time points during drought stress of maize plant roots. **(A)** Twenty representative profiles of different expression trends. The colored background parts showed significance, and the genes of the same type were gathered in the same cluster. **(B, C)** The most significantly enriched GO terms of DEMRs from the two comparison groups. **(D, E)** The most significantly enriched KEGG pathway of DEMRs from the two comparison groups.

To investigate the expression pattern of the 3,765 DEMRs under drought stress, their fragments per kilobase of transcript per million mapped reads (FPKM) values in roots were normalized, then analyzed using Short Time-series Expression Miner (STEM). Sixteen model profiles were constructed, eight of which were significant. Gene expression profiles are illustrated in **Figure 3A**; areas with colored backgrounds indicate significant differences, and genes of the same type are clustered. Profile 12 was significantly enriched in roots, with the smallest p-value; therefore, our subsequent research work may focus on genes in profile 12.

To further study the function of DEMRs in maize root, we annotated them into three categories (biological process, cellular component, and molecular function) using the GO database. Under drought stress, WT_vs_DSSD root DEMRs were mainly enriched in biological processes, including response to oxygen-containing compound (GO:1901700), response to lipid (GO:0033993), response to abscisic acid (GO:0009737), and response to alcohol (GO:0097305); cellular components comprising plasma membrane part (GO:0044459), intrinsic component of plasma membrane (GO:0031226), plant-type cell wall (GO:0009505), and integral component of plasma membrane (GO:0005887); and molecular functions, including receptor serine/threonine kinase binding (GO:0033612), monovalent inorganic cation transmembrane transporter activity (GO:0015077), potassium ion transmembrane transporter activity (GO:0015079), and microtubule binding (GO:0008017). Comparison of the WT_vs_DSFD groups under stress indicated that root biological processes were mainly enriched for transmembrane transport (GO:0055085), response to high light intensity (GO:0009644), response to hydrogen peroxide (GO:0042542), and ion transport (GO:0006811); cellular components were enriched for intrinsic component of plasma membrane (GO:0031226), integral component of plasma membrane (GO:0005887), plasma membrane part (GO:0044459), and intrinsic component of membrane (GO:0031224), while enriched molecular functions included transmembrane transporter activity (GO:0022857), transporter activity (GO:0005215), inorganic molecular entity transmembrane transporter activity (GO:0015318), and carbohydrate transmembrane transporter activity (GO:0015144) (**Figures 3B, C**).

To further explore the similarities and differences in DEMR functional enrichment, KEGG enrichment analysis was performed for the 3,765 DEMRs and 20 pathways. The results showed that drought stress-altered DEMRs in the WT_vs_DSSD and WT_vs_DSFD groups were concentrated in pathways including phenylpropanoid biosynthesis (ko00940), plant hormone signal transduction (ko04075), starch and sucrose metabolism (ko00500), and glutathione metabolism (ko00480) (**Figures 3D, E**). The remaining eight pathways were enriched for DEMRs, but not for comparisons of both the WT_vs_DSSD and WT_vs_DSFD groups.

### Analysis of co-expression networks and qRT-PCR validation of relationships between DEMIRs and DEMRs

**Figure 4:**
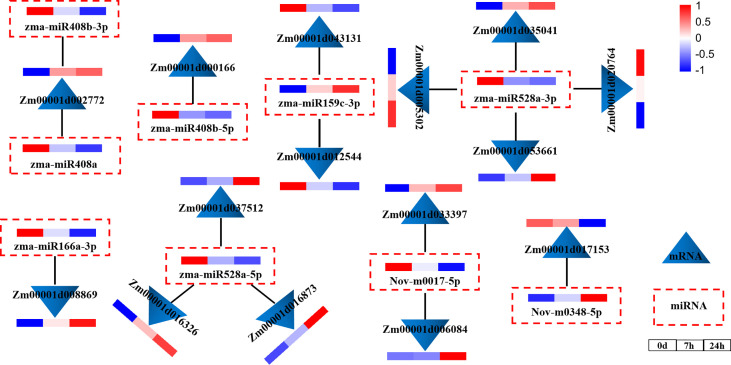
miRNA and mRNA co-expression network diagram. Network analysis was performed using the Cytoscape network platform.

**Figure 5:**
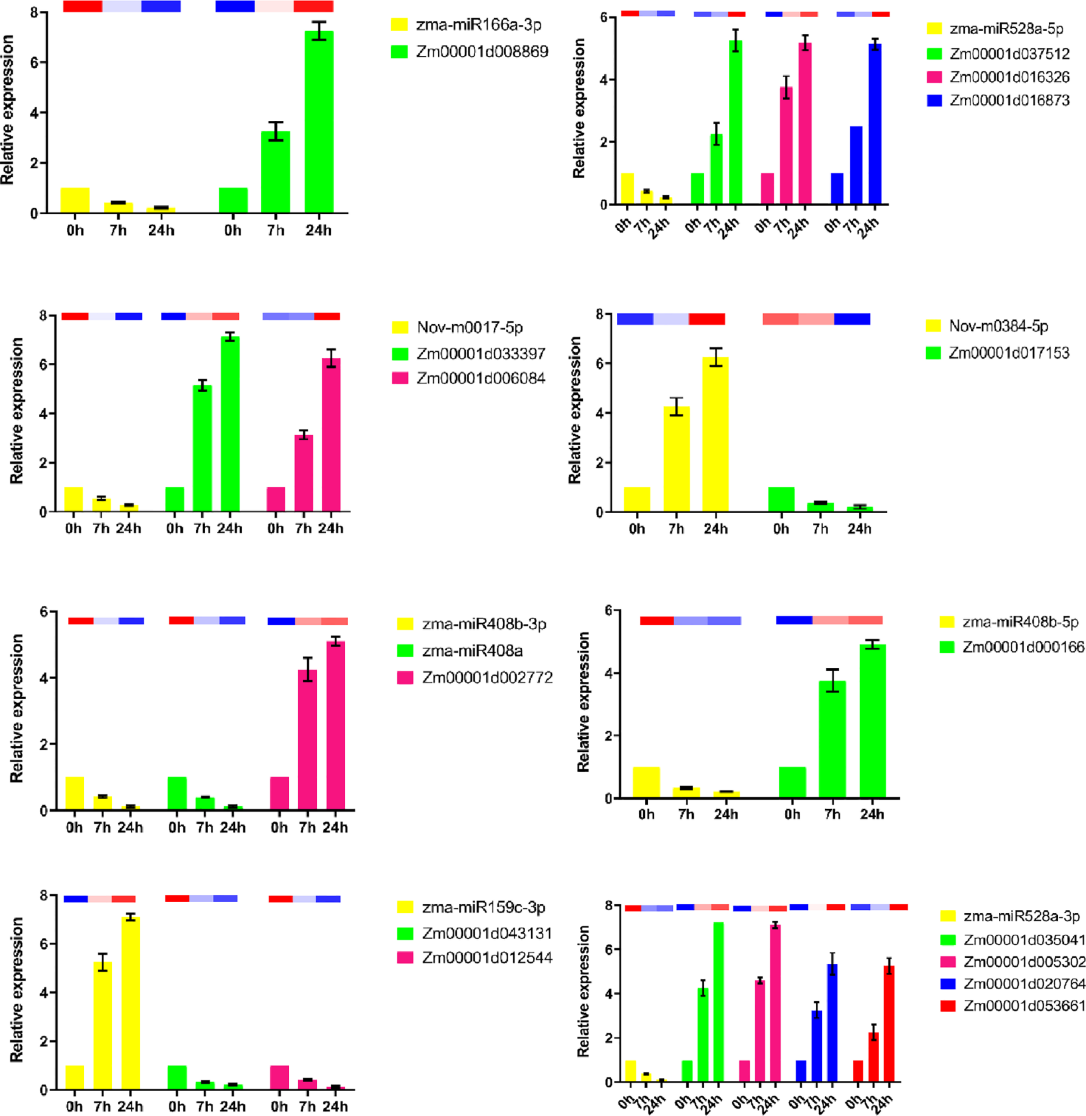
qRT-PCR analysis of DEMIs and DEMs in maize roots under drought stress. The 2^-ΔΔCt^ was used to calculate the fold change of expression in qRT-PCR analysis, with U6 and Actin as reference for miRNA and target genes, respectively. All experiments were repeated three times, and the expression data were log2 transformed before analysis. The error line is the standard error. The column charts represent qPCR data and heatmaps represent RNA-Seq data.

To explore the regulatory relationship between miRNAs and mRNAs under drought stress, we constructed a molecular regulatory network diagram of DEMIRs and DEMRs (**Figure 4**), which showed that 9 DEMIRs negatively regulated the expression of 15 DEMRs. The reliability of the transcriptome data was verified by qRT-PCR analysis. Based on those with high FPKM and multiple changes, the expression patterns of 9 DEMIRs and 15 DEMRs closely related to drought stress response were verified at 0, 7, and 24 h. The results showed that the expression patterns determined by qRT-PCR and RNA-seq were consistent, confirming the accuracy of the RNA-seq data (**Figure 5**).

### Analysis of key pathways in maize responses to drought stress

**Figure 6:**
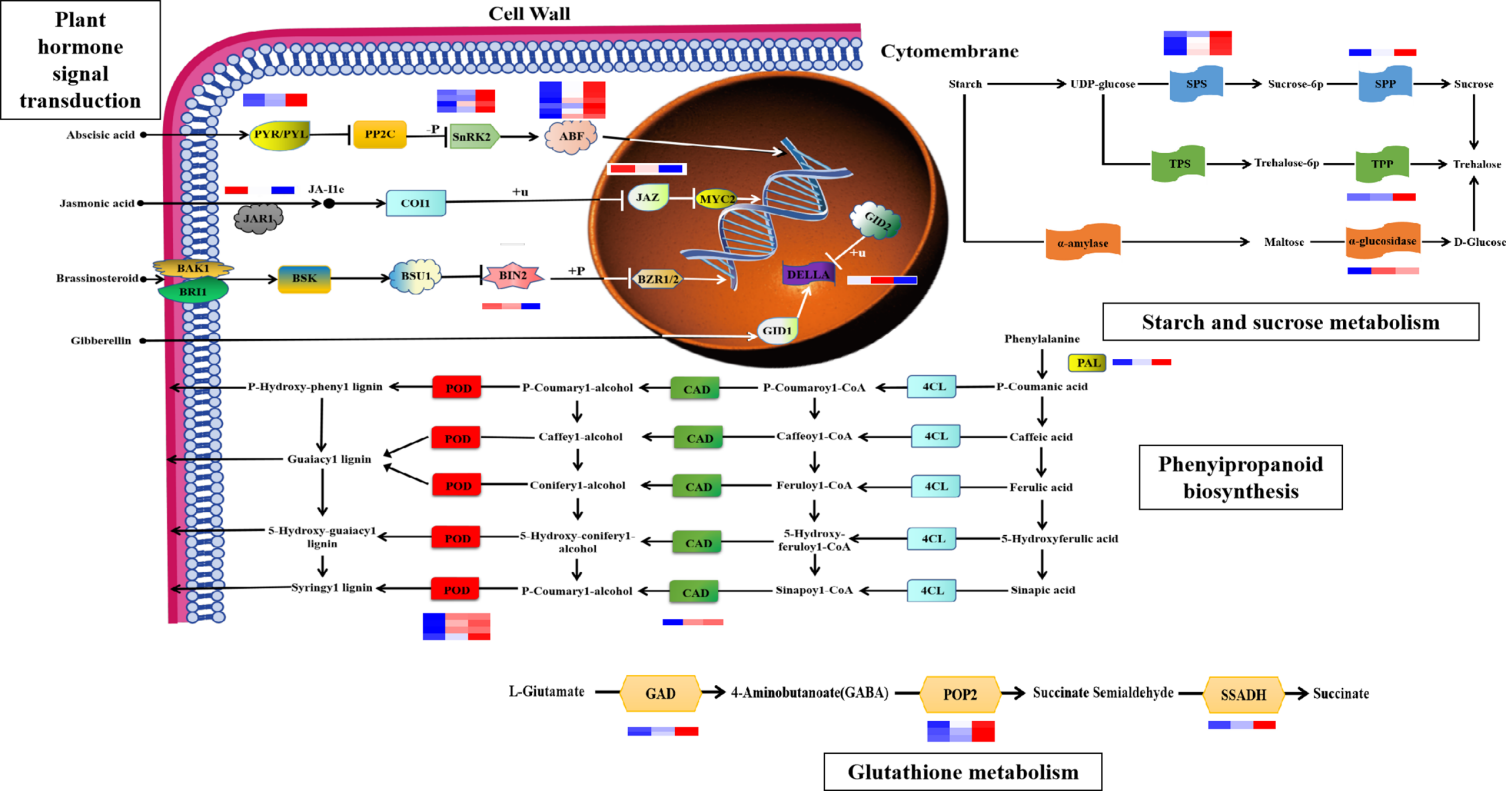
The key pathways of the drought stress response in maize. These pathways were “plant hormone signal transduction”, “phenylpropanoid biosynthesis”, “starch and sucrose metabolism”, and “glutathione metabolism”.

Based on biological characteristics and gene functions, we identified four pathways important for the maize root system response to drought stress (**Figure 6**, **Supplementary Table S1**), including: “plant hormone signal transduction”, “phenylpropanoid biosynthesis”, “glutathione metabolism”, and “starch and sucrose metabolism”. Molecules involved in “hormone signal transduction” pathways, including ABA receptors, PYR/PYL (Zm00001d012475 and Zm00001d010445); positive regulator phosphokinase, SnRK2 (Zm00001d042695, Zm00001d013736, Zm00001d029975, and Zm00001d033339); and the downstream regulator of ABA signaling, ABF (Zm00001d012296, Zm00001d044940, Zm00001d020711, Zm00001d042721, Zm00001d050018, Zm00001d031790, and Zm00001d018178), were all upregulated in the maize root system after drought stress, which would be expected to enhance ABA signal transduction. Both the jasmonate amino acid conjugating enzyme, JAR1 (Zm00001d008957), and the JA signaling hub protein, JAZ (Zm00001d024455), were downregulated in response to drought, which would be predicted to attenuate JA signal transduction. BIN2 (Zm00001d053548), a negative regulator of the BR signaling pathway, is a type of protein kinase whose expression is downregulated under drought stress and negatively correlated with BR content. The DELLA protein is a negative regulator of the gibberellin signal transduction pathway, and a nuclear protein involved in growth inhibition. DELLA protein (Zm00001d044065) expression was upregulated 0–7 h after drought stress, and negatively correlated with GA3 content. In the starch and sucrose metabolic pathways, expression of the sucrose phosphate synthase (SPS) (Zm00001d012036, Zm00001d042353, Zm00001d048979, and Zm00001d050125) and sucrose phosphate phosphatase (Zm00001d010523) genes was upregulated following drought stress. Expression of genes related to trehalose 6-phosphate phosphatase (TPP) (Zm00001d006913) and α-glucosidase (Zm00001d036608) was also upregulated, and trehalose content was positively correlated with expression of these mRNAs. In the “glutathione metabolism” pathway, mRNA expression levels of glutamate decarboxylase (GAD) (Zm00001d031749 and Zm00001d033805), alanine aminotransferase (POP2) (Zm00001d049380, Zm00001d015444, and Zm00001d037507), and succinic semialdehyde dehydrogenase (SSADH) (Zm00001d015406) were all upregulated after drought stress. In the “phenylalanine biosynthesis” pathway, the expression levels of genes related to phenylalanine ammonia lyase (PAL) (Zm00001d051163), cinnamyl alcohol dehydrogenase (CAD) (Zm00001d015618), and peroxidase (Zm00001d048413, Zm00001d038599, Zm00001d009140, and Zm00001d014606) were all upregulated in the maize root system following drought stress.

### Drought stress reduces the accumulation of ZmmiR408a, which negatively regulates drought tolerance in maize

**Figure 7:**
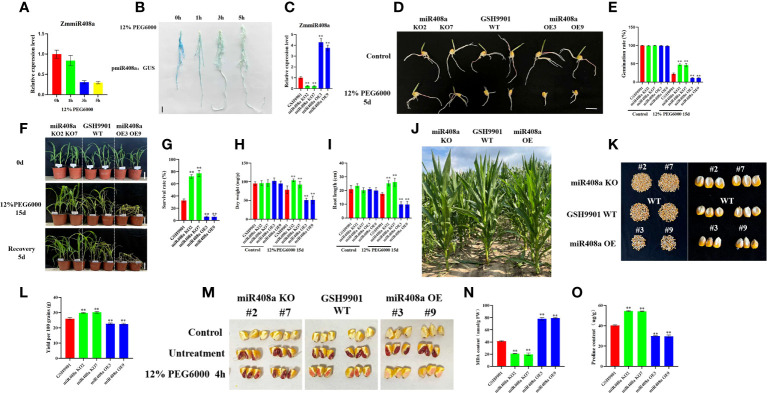
Analysis of phenotype and physiological and biochemical indexes of ZmmiR408a transgenic plants under drought stress. **(A)** Expression pattern analysis of ZmmiR408a in roots upon drought treatment. **(B)** Histochemical analysis of ZmmiR408a promoter: GUS transgenic maize roots were done under 0-h, 1-h, 3-h, and 5-h drought treatment. **(C)** Real-time quantitative PCR analysis of the expression level of ZmmiR408a gene in miR408a-OE and miR408a-KO plants. **(D, E)** Seed germination phenotypes of transgenic lines and wild-type plants under non-treatment and 12% PEG6000 treatment for 5 days. The scale bar represents 2 cm. **(F, G)** Analysis of wilting degree and recovery ability of transgenic lines and wild-type plants under drought treatment for 15 days and recovery for 5 days. All the plants were grown in a greenhouse at 25 ± 2°C under a 16-h light/8-h dark photoperiod. **(H)** Dry weight of wild-type, miR408a-KO, and miR408a-OE seedlings under normal and drought stress conditions. **(I)** Root length of wild-type, miR408a-KO, and miR408a-OE plants recorded after 15 days of non-treatment and drought treatment. **(J)** Phenotypes of plant height of wild-type, miR408a-KO and miR408a-OE plants under drought treatment in the field. **(K, L)** Grain phenotype and 100-seed weight analysis of wild-type, miR408a-KO, and miR408a-OE plants under drought treatment. **(M)** Seed vigor of wild-type, miR408a-KO and miR408a-OE plants under 12% PEG6000 treatment. A total of 0.4% TTC solution was used for staining observation of seed vigor. Control: maize kernels boiled in boiling water for 2 h; Untreatment: maize kernels without drought treatment. **(N, O)** Malondialdehyde (MDA) content and Proline (Pro) content in leaves of wild-type, miR408a-KO, and miR408a-OE plants under drought treatment. Data were expressed as the mean of triplicate values and error represented the SD, p < 0.05 (*) and p < 0.01 (**).

Our analysis of miR408a expression, based on stem-loop RT-qPCR, demonstrated that ZmmiR408a was significantly downregulated in roots in response to drought treatment (**Figure 7A**); therefore, we conducted GUS-staining of pmiR408a:GUS. Levels of the miR408a-driven GUS gene were significantly reduced in the root system on increasing duration of exposure to 12% PEG6000-induced drought (**Figure 7B**). We further confirmed the consistency of ZmmiR408a expression patterns in the inbred line, GSH9901, under drought stress conditions.

To investigate the role of ZmmiR408a in drought responses, two independent ZmmiR408a gene-edited plant lines, miR408a KO2 and miR408a KO7, and two independent ZmmiR408a overexpressing plant lines, miR408a OE3 and miR408a OE9, were generated. Fluorescence quantitative PCR showed that miR408a expression was significantly decreased in miR408a KO plants, while it was significantly increased in miR408a OE plants (**Figure 7C**). Under normal conditions, there was no significant difference in seed germination between transgenic and WT strains; however, very interestingly, the seed germination ability of the miR408a KO strain was significantly improved compared with that of the WT and miR408a OE strains under treatment with 12% PEG 6000 stress for 5 days (**Figures 7D, E**). Furthermore, after 15 days of drought stress, leaf wilting degree (**Figure 7F**), plant survival rate (**Figure 7G**), dry weight (**Figure 7H**), and root length (**Figure 7I**) were superior in the miR408a KO strain than those of WT and miR408a OE strains. In maize fields under drought conditions, the height of transgenic strains did not differ significantly from that of WT plants (**Figure 7J**), while seed setting rate (**Figure 7K**), yield (**Figure 7L**), and seed vigor (**Figure 7M**) were significantly better in the miR408a KO strain than those of the WT and miR408a OE strains. Physiological analysis showed that, under drought treatment, the MDA content of the miR408a KO strain was lower than that of WT and miR408a OE strains (**Figure 7N**), while the proline content was higher (**Figure 7O**). In conclusion, these results show that ZmmiR408a is a negative regulator of drought tolerance.

### ZmmiR408a regulates drought tolerance by modulating ROS accumulation in maize

**Figure 8:**
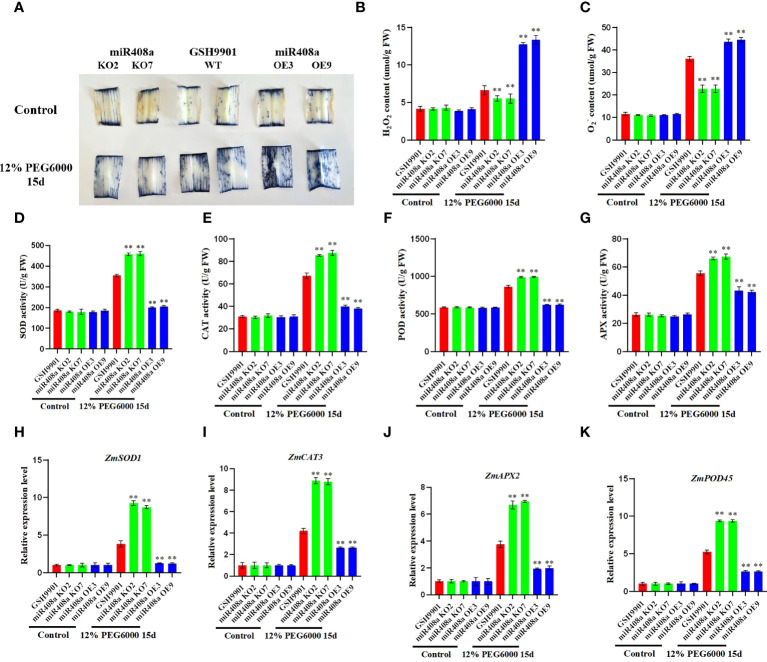
ZmmiR408a reduced drought tolerance by regulating ROS accumulation in maize. **(A)** Nitrotetrazolium Blue chloride (NBT) staining of wildtype, miR408a-KO, and miR408a-OE plants were conducted drought treatment for 15 days, which was used to monitor the ROS production in drought-treated leaves. **(B, C)** Hydrogen peroxide (H_2_O_2_) content and Superoxide radical (O_2_^−^) content in leaves. **(D–G)** Analysis of catalase (CAT), superoxide dismutase (SOD), peroxidase (POD), and ascorbate peroxidase (APX) activity in drought-treated leaves. **(H–K)** Analysis on expression patterns of ROS-related Marker genes in drought treated leaves. The expression level was normalized to that of Maize Actin. Data were expressed as the mean of triplicate values and error represented the SD, p < 0.05 (*) and p < 0.01 (**).

To investigate whether ZmmiR408a can regulate ROS accumulation, we conducted NBT staining to evaluate O_2_^−^ levels. Notably, under drought treatment, NBT staining intensity in the leaves of the miR408a-KO strain was significantly lower than that in the WT and miR408a-OE strains (**Figure 8A**). Furthermore, the accumulation and production of H_2_O_2_ and O_2_^−^ in leaves from WT, miR408a-KO, and miR408a-OE maize seedlings were compared. Under normal growth conditions, there was no significant difference in H_2_O_2_ and O_2_^−^ contents between the transgenic and WT strains; however, following 15 days of drought treatment using 12% PEG 6000, H_2_O_2_ and O_2_^−^ contents in the leaves of the miR408a-KO strain were significantly lower than those in WT and miR408a-OE strain leaves (**Figures 8B, C**). Jiao et al. (2022a) found that antioxidant enzymes can effectively scavenge high concentrations of ROS. Therefore, SOD, CAT, peroxidase, and APX activities in WT and transgenic plant leaves were quantitatively analyzed. Under normal conditions, no significant difference in antioxidant enzyme activity was detected between WT, miR408a-KO, and miR408a-OE strains; however, under drought treatment, the miR408a-KO strain had the highest enzyme activity in its leaves (**Figures 8D–G**). Expression levels of ROS homeostasis-related genes (ZmSOD1, ZmCAT3, ZmPOD45, and ZmAPX2) were detected by real-time fluorescence quantitative PCR. Under normal conditions, there was minimal difference between levels in WT, miR408a-KO, and miR408a-OE strains. By contrast, ROS scavenging-related genes were all upregulated in WT, miR408a-KO, and miR408a-OE plants under drought stress, and those in miR408a-KO plants were consistently higher than corresponding levels in WT and miR408a-OE plants (**Figures 8H–K**). These results show that effective knockout of miR408a genes can improve SOD, APX, and CAT enzyme activities, as well as ROS-related gene expression, under drought conditions, thereby reducing ROS accumulation.

## Discussion

Drought stress severely restricts the growth and development of corn plants in the field and is among abiotic stress factors important for yield loss (Guo et al., 2021). When grown under drought conditions, the roots of most plants change significantly (Chen et al., 2020); therefore, topics related to the response of the maize root system to drought resistance have become the focus of intense research. In this study, the morphological and physiological differences of maize seedlings under drought stress were investigated and a series of DEMIRs and DEMRs in the maize seedling root system in response to drought were identified. Furthermore, an miRNA–mRNA network was constructed and four key pathways were explored. Based on our findings, we also investigated the biological function of miR408a in the maize root system, providing a new strategy for further exploration of the mechanisms underlying maize root system responses to drought stress.

### MiRNA sequencing analysis and drought stress response

Abiotic stress generates marked molecular responses that influence the healthy growth and development of plants. In recent years, the molecular mechanisms underlying plant responses to abiotic stress have attracted extensive attention (Alcázar et al., 2006; Shi and Chan, 2014; Luan et al., 2015; Ansari and Ahmad, 2019; Zhou et al., 2021). To investigate the molecular mechanisms involved in maize seedling root system responses to drought stress, we performed sRNA sequencing analysis. Data analysis identified 32 DEMIRs from 9 libraries, of which 25 known miRNAs were from 9 miRNA families (**Table S2**). Among these miRNA families, miR159, miR393, miR169, miR390, miR528, miR397, miR398, and miR166 have been shown to have roles in drought stress responses of other plants (Jagadeeswaran et al., 2009; Hamza et al., 2016; Lu et al., 2018; Zhang et al., 2018; López-Galiano et al., 2019; Yuan et al., 2019; Li et al., 2021a; Chen et al., 2021). Nevertheless, there are few relevant studies into the regulation of root drought resistance by the miR408 family. Zhang et al. (2017) found that miRNA408 in rice can affect the homeostasis of copper ions in chloroplasts by mediating target genes, resulting in enhanced stress resistance, enhanced photosynthetic rate, and yield. Ma et al. (2015) found that under the effects of low temperature, drought, oxidation, and osmotic pressure, miR408 in Arabidopsis thaliana mediated the target gene CSD1 to improve plant stress resistance and oxidative stress response. Therefore, we preliminarily conducted verification of the biological function of maize root system miR408a in response to drought stress. In addition, we predicted numerous new miRNAs, seven of which were DEMIRs (**Supplementary Table S2**), and a future study of their biological functions in plant abiotic stress will be of considerable interest.

### mRNA sequencing analysis and drought stress response

Transcriptome sequencing analysis of the maize root system under drought stress identified 1,846 upregulated and 1,919 downregulated DEGs. Based on sequencing data and KEGG enrichment analysis, the 3,765 DEGs were found to be mainly enriched in four pathways: “plant hormone signal transduction”, “phenylpropane biosynthesis”, “glutathione metabolism”, and “starch and sucrose metabolism”. This result was consistent with those of the current study, because differences in relevant biological data suggested that these four pathways may be involved in responses to drought stress. Kumar et al. (2020) found that DEGs related to plant hormone signal transduction are regulated under drought stress in mung beans, while Yu et al. (2020) found that DEGs related to phenylpropanoid biosynthesis have important regulatory roles under drought stress in foxtail millet. Furthermore, Song et al. (2021) found that japonica and indica rice exhibit strongly DEGs related to the glutathione metabolic pathway under water shortage conditions, while Qian et al. (2021) found that the expression of DEGs related to starch and sucrose metabolic pathways was increased under drought conditions, indicating their importance in stress tolerance. These findings, together with those of this study, confirm that “plant hormone signal transduction”, “phenylpropane biosynthesis”, “glutathione metabolism”, and “starch and sucrose metabolism” are important pathways in maize responses to drought stress, which further supports the findings of the current study.

### MiRNA–mRNA regulatory network plays a key role in plant responses to abiotic stresses

Kang et al. (2021) and Yang et al. (2021a) demonstrated that combined analysis of miRNA–mRNA can reveal relevant regulatory mechanisms with important roles in plant responses to abiotic stress. Here, we conducted combined analysis of miRNAs and mRNAs related to maize root system drought stress responses, detected correlations between a series of DEMIRs and DEMRs, and constructed a regulatory network map. Many studies have reported miRNA target genes related to plant hormone signal transduction (Li et al., 2019; Cui et al., 2020; Singh et al., 2021; Lei et al., 2021; Yang et al., 2021b). Most interestingly, we also found that numerous target genes regulated by miRNAs were also enriched in the three pathways: “phenylpropane biosynthesis”, “glutathione metabolism”, and “starch and sucrose metabolism”. Discovery of these target genes lays a theoretical foundation for follow-up research into the biological functions of miRNAs regulated in the maize root system under drought stress.

### Plant hormone signal transduction pathway is a crucial process in regulating environmental stress responses

Blázquez et al. (2020) proposed that, with developments in high-throughput sequencing and new gene manipulation techniques, various plant hormones with important roles in regulating environmental stress responses will be identified. As an important plant hormone, ABA functions in response to abiotic stressors (such as drought, saline, alkali, and cold), as well as in plant growth and development. Since abiotic stress affects crop yield, study of ABA signaling in plants can effectively improve crop quality. Overexpression of the ABA receptors, PYR/PYL, can effectively inhibit protein phosphatase 2C (PP2C) and release SnRK2 protein kinases during ABA signal transduction, and further activate ABF transcription factor-mediated regulation of downstream target genes. Here, we found that the PYR/PYL-PP2Cs-SnRK2s regulatory module was activated, and that related protein-coding genes were upregulated, thus triggering ABA signaling. Based on analysis of physiological indices, we confirmed that increased ABA content in the maize root system was positively correlated with expression of PYR/PYL, SnRK2s, and ABF-related genes.

JA molecules are a ubiquitous class of adipose hormones that regulate plant growth and development, as well as stress responses. Li et al. (2021b) found that the COI1–JAZ complex is a JA pathway receptor, which mediates JAZ degradation and thereby influences JA pathway transcription factors to regulate downstream target gene expression. In this study, we found that the jasmonate amino acid conjugating enzyme, JAR1, and the JA signaling hub protein, JAZ, were downregulated and, in turn, attenuated JA signal transduction, which was positively correlated with physiological changes in JA content.

BRs are a class of hormones with crucial roles in plant growth and development. Jiang et al. (2019) found that the negative regulator of BRs signaling, BIN2, can promote the upregulation of RD26 genes to improve plant drought resistance. Lin et al. (2013) found that miR395a regulated root growth and stress tolerance of Arabidopsis seedlings by inhibiting GUN5 expression and its downstream signal transduction under brassinolide treatment. Here, we found that BIN2 was significantly downregulated in response to increased drought duration, and negatively correlated with physiological alterations in BR content.

GA3 is a growth-regulating hormone involved in stress responses. Zheng et al. (2020) confirmed that miR396b targets SmGRFs, SmHDT1, and SmMYB37/4 to mediate the phytohormone, especially gibberellin signaling pathways and consequentially resulted in the phenotype variation of miR396b-OE hairy roots. Furthermore, miR396b could be activated by methyl jasmonate, abscisic acid, gibberellin, salt, and drought stresses. In this study, we found that decreased GA3 content with increased drought duration could affect the expression of DELLA genes.

Together, these findings show that plant hormones have important regulatory roles in responses to abiotic stress, that regulation of multiple hormones is a likely focus of intense plant research, and that the ABA, JA, BRS, and GA3 signaling pathways may be involved in maize root system-mediated drought tolerance.

### Phenylpropanoid biosynthesis pathway affects plant resistance to stress

The phenylpropanoid biosynthesis pathway can affect stress responses, as well as plant growth and development. Dong and Lin (2021) found that the plant phenylpropane metabolic pathway, particularly lignin synthesis, has important regulatory functions in plant responses to biotic and abiotic stressors. The initial reaction of phenylpropane metabolism involves PAL, while CAD genes are important for lignin biosynthesis. Sun et al. (2020) found that overexpression of the cotton Gh4CL7 gene in Arabidopsis can increase the expression of the lignin synthesis gene PAL, thereby improving drought resistance. Liu et al. (2020) found that overexpression of CmCAD2 and CmCAD3 genes could improve drought tolerance through recovering lignin synthesis and root development in Arabidopsis. Here, we conducted deep analysis of the reasons underlying significant upregulation of PAL genes with increased drought duration, which in turn leads to significant elevation of CAD gene expression and activation of the phenylpropane biosynthesis pathway. Peroxidase genes function in the final pathway of “phenylpropane biosynthesis”. Here, we demonstrated that CAD genes were activated with increased drought duration, followed by activation of peroxidase genes, leading to lignin accumulation and improved maize drought resistance, suggesting that lignin synthesis in the root system is an important feature of maize responses to drought stress.

### Glutathione metabolic pathway plays an important resistance system in plant signal transduction and stress responses

Glutathione metabolism is important for plant responses to biotic and abiotic stress. Nerva et al. (2022) showed that expression of glutathione-S-transferase can effectively improve grape drought tolerance, and activate the expression of antioxidants and ABA-related transcripts. Priya et al. (2019) found that GABA genes can increase the activity of respiratory-related enzymes under heat stress in mung beans, thus protecting plant growth and development. Jin et al. (2019) found that exogenous application of GABA genes can effectively improve muskmelon tolerance to hypoxic stress and avoid photooxidative plant damage. Here, we showed that GAD gene expression was upregulated in the maize root system with increased drought duration, which, in turn, promoted the GABA gene expression. Furthermore, mRNA levels of four POP2-related genes were significantly increased, which promoted SSADH gene expression, and finally activated the glutathione metabolic pathway.

### Starch and sucrose metabolic pathways are vital mechanisms in regulating the physiological mechanisms of plant abiotic stress

Starch and sucrose metabolism are important mechanisms in regulation of plant responses to drought stress. Wang et al. (2020) found that expression of key genes related to starch and sucrose metabolism is induced under long-term drought stress in tobacco. Moenga et al. (2020) conducted transcriptome analysis of wild and cultivated chickpeas under drought stress and found that some transcripts involved in starch and sucrose transport were activated. SPS is the key rate-limiting enzyme in sucrose anabolism, and Li et al. (2011) found that overexpression of the TPP gene in rice can effectively improve drought resistance. Furthermore, Nuccio et al. (2015) found that overexpression of TPP genes can promote trehalose metabolism to generate sucrose and improve potato drought tolerance in the greenhouse. In this study, we showed that SPS and TPP genes were significantly upregulated with increased drought duration, consistent with the results of the published studies mentioned above. Gupta et al. (2020) found that drought signaling can affect the generation of metabolites, such as trehalose and proline, stimulate the antioxidant system to maintain redox homeostasis, and prevent cell damage and disruption of cell membrane integrity, through oxide enzyme activity. Here, we found that TPP was a key enzyme in trehalose synthesis and that trehalose content increased rapidly in the maize root system with increased duration of drought. Therefore, upregulation of trehalose biosynthesis and trehalose accumulation may have protective and regulatory effects on the maize root system under drought stress.

## Conclusions

Drought stress has significant effects on maize plant performance. ABA, BRs, GSH, lignin, and trehalose content, as well as peroxidase activity, increased, while GA and JA content decreased with increased drought stress duration. A total of 32 DEMIRs and 3,765 DEMRs were identified, and 16 miRNA–target gene pairs, composed of 9 DEMIRs and 15 DEMRs, were obtained by miRNA–mRNA combined analysis. Four important metabolic pathways, “plant hormone signal transduction”, “phenylpropane biosynthesis”, “glutathione metabolism”, and “starch and sucrose metabolism”, were analyzed. Additionally, we demonstrate that miR408a is a negative regulator of drought response in the maize root system, primarily through regulation of ROS accumulation.

## Data availability statement

The datasets presented in this study can be found in online repositories. The data presented in the study are deposited in the NCBI repository, accession number PRJNA796152 and PRJNA793522.

## Author contributions

SG and YM conceived research plans and designed experiments. PJ, CW, and NC conducted experiments. PJ and RM wrote the draft. SL and JQ analyzed the data. PJ, SG, and YM reviewed and edited this article and provided helpful comments and discussions. All authors read and approved the final manuscript.

## Funding

This work was supported by Jilin Province Science and Technology Development Plan Project (20200402023NC and 20210302003NC). Furthermore, the funding body has no role in designing the study, collection and interpretation of data, or writing the manuscript.

## Conflict of interest

The authors declare that the research was conducted in the absence of any commercial or financial relationships that could be construed as a potential conflict of interest.

## Publisher’s note

All claims expressed in this article are solely those of the authors and do not necessarily represent those of their affiliated organizations, or those of the publisher, the editors and the reviewers. Any product that may be evaluated in this article, or claim that may be made by its manufacturer, is not guaranteed or endorsed by the publisher.
