# Supplementary material for: Integration of mRNA and microRNA analysis reveals the molecular mechanisms underlying drought stress tolerance in maize (Zea mays L.)
Source: Front Plant Sci. 2022 Sep 29;13:932667. doi: 10.3389/fpls.2022.932667 (PMC9557922; doi:10.3389/fpls.2022.932667)
Supplement: Supplementary file 1 [file DataSheet_1.doc]

Table S1 Gene expression in the pathway

| desc | ID | 0 h | 7 h | 24 h |
| --- | --- | --- | --- | --- |
| POD | Zm00001d048413 | 1.34 | 3.31 | 3.32 |
|  | Zm00001d038599 | 28.65 | 52.68 | 59.9 |
|  | Zm00001d009140 | 16.34 | 67.75 | 71.36 |
|  | Zm00001d014606 | 3.43 | 21.21 | 50.09 |
| CAD | Zm00001d015618 | 289.39 | 465.08 | 481.88 |
| PAL | Zm00001d051163 | 52.29 | 79.18 | 120.51 |
| GID1 | Zm00001d010308 | 26.74 | 35.24 | 26.39 |
| DELLA | Zm00001d044065 | 2.68 | 3.8 | 1.76 |
| PYR/PYL | Zm00001d012475 | 0.03 | 0.14 | 0.47 |
|  | Zm00001d010445 | 63.62 | 67.86 | 84.23 |
| SnRk | Zm00001d042695 | 42.9 | 63.31 | 175.4 |
|  | Zm00001d013736 | 18.87 | 19.28 | 39.92 |
|  | Zm00001d029975 | 57.43 | 119.12 | 149.44 |
|  | Zm00001d033339 | 21.37 | 32.03 | 63.19 |
| ABF | Zm00001d012296 | 0.02 | 0.21 | 0.41 |
|  | Zm00001d044940 | 8.17 | 25.88 | 44.31 |
|  | Zm00001d020711 | 16.66 | 29.97 | 42.66 |
|  | Zm00001d042721 | 14.51 | 27.66 | 33.66 |
|  | Zm00001d050018 | 4.12 | 6.67 | 17.56 |
|  | Zm00001d031790 | 14.53 | 32.65 | 52.41 |
|  | Zm00001d018178 | 9.26 | 38.47 | 44.69 |
| JAR1 | Zm00001d008957 | 25.76 | 17.64 | 9.56 |
| JAZ | Zm00001d024455 | 15.64 | 12.33 | 7.1 |
| BIN2 | Zm00001d053548 | 62.48 | 60.34 | 45.99 |
| GAD | [Zm00001d031749](http://plants.ensembl.org/Zea_mays/Gene/Summary?g=Zm00001d047981) | 41.23 | 196.93 | 703.43 |
|  | Zm00001d033805 | 47.54 | 56.5 | 72.57 |
| SSADH | Zm00001d015406 | 14.23 | 52.65 | 161.69 |
| POP2 | Zm00001d049380 | 49.42 | 113.7 | 186.75 |
|  | Zm00001d015444 | 0.7 | 0.83 | 1.23 |
|  | Zm00001d037507 | 12.72 | 15.82 | 32.02 |
| SPS | Zm00001d012036 | 0 | 0.053 | 0.34 |
|  | Zm00001d042353 | 0.91 | 2.32 | 3.66 |
|  | Zm00001d048979 | 8.15 | 16.58 | 23.89 |
|  | Zm00001d050125 | 35.81 | 66.21 | 86.13 |
| SPP | Zm00001d010523 | 28.55 | 45.14 | 64.37 |
| TPP | Zm00001d006913 | 0.34 | 0.37 | 0.68 |
| α-glucosidase | [Zm00001d036608](http://plants.ensembl.org/Zea_mays/Gene/Summary?g=Zm00001d036608) | 23.08 | 39.81 | 36.67 |

Table S2 Differential expression miRNA TPM value and type

| ID | Drought-0h TPM | Drought-7h TPM | Drought-24h TPM | Type |
| --- | --- | --- | --- | --- |
| zma-miR159c-3p | 80562 | 128669 | 153411 | up |
| zma-miR159d-3p | 80562 | 128669 | 153411 | up |
| zma-miR393b-3p | 4640 | 9170 | 8754 | up |
| zma-miR169c-3p | 884 | 199 | 215 | down |
| zma-miR390a-5p | 976 | 322 | 401 | down |
| zma-miR390b-5p | 976 | 322 | 401 | down |
| zma-miR408b-5p | 397 | 63 | 23 | down |
| zma-miR528a-3p | 263 | 33 | 9 | down |
| zma-miR528b-3p | 263 | 33 | 9 | down |
| zma-miR397a-5p | 12218 | 4842 | 1748 | down |
| zma-miR397b-5p | 12218 | 4842 | 1748 | down |
| zma-miR528a-5p | 5913 | 1829 | 680 | down |
| zma-miR528b-5p | 5913 | 1829 | 680 | down |
| zma-miR398a-5p | 166 | 43 | 9 | down |
| zma-miR408a | 10149 | 5143 | 2119 | down |
| zma-miR408b-3p | 10149 | 5143 | 2119 | down |
| zma-miR166c-3p | 24506 | 13816 | 6869 | down |
| zma-miR166d-3p | 24506 | 13816 | 6869 | down |
| zma-miR166a-3p | 24506 | 13816 | 6869 | down |
| zma-miR166e | 24506 | 13816 | 6869 | down |
| zma-miR166f | 24506 | 13816 | 6869 | down |
| zma-miR166i-3p | 24506 | 13816 | 6869 | down |
| zma-miR166h-3p | 24506 | 13816 | 6869 | down |
| zma-miR166g-3p | 24506 | 13816 | 6869 | down |
| zma-miR166b-3p | 24506 | 13816 | 6869 | down |
| Nov-m1141-3p | 1 | 84 | 14 | up |
| Nov-m0015-5p | 0 | 19 | 10 | up |
| Nov-m0052-5p | 0 | 17 | 10 | up |
| Nov-m0348-5p | 0 | 7 | 19 | up |
| Nov-m0987-3p | 31 | 15 | 0 | down |
| Nov-m0017-5p | 26 | 12 | 0 | down |
| Nov-m1122-3p | 19 | 9 | 0 | down |

Table S3 Gene IDs and primer sequences for the genes used for qPCR verification.

| Gene ID | Forward primer | Reverse primer |
| --- | --- | --- |
| Zm00001d002772 | CGGCCGTGCATATTTTTCGT | TCGAGTCCTCTGTTTGCCAC |
| Zm00001d000166 | CTGTTCTGGGAACGTCGGAA | TCCCAAAGAATCATGGGGGC |
| Zm00001d043131 | TGAGCGATGCCATTGACAGT | TCTTGCTGCTAGCATTGGCT |
| Zm00001d012544 | TGATTTGAACGGTGGCGAGA | GGGTCGCTGATAGAGACTGC |
| Zm00001d035041 | GAAGATGCAGCAGCTAACGC | GCGCATCGCCATTTTGGTAT |
| Zm00001d005302 | GTGCCCAGAAGGCCAACTAT | TCCCCTTGTTGCCCCTTTTT |
| Zm00001d053661 | GGTCTTCCCCTAACCGCTTC | CCAGCTCTGTCACACGTCAT |
| Zm00001d020764 | GCACAACAACTCACGGTGTC | CGACCGATCCTGCAGCTTAT |
| Zm00001d008869 | TCCCCGAGTAGAGTGGTCAG | CATCGCAGAAGCGAAGAACG |
| Zm00001d037512 | GGATTTGATGCCTTCGCGTC | GAAGCCCTCCCCATATTCCG |
| Zm00001d016326 | TCTTTTGCTTCTTTGCCGCC | AGGCCTACGGTAGTGGCTAA |
| Zm00001d016873 | TCGTTCCGAACTCCAACAGG | GCTCGCGTGACGTGATTTAC |
| Zm00001d033397 | GTGACAGCTTGGGGTGCTAT | CAGACCCAAACAAGCACACG |
| Zm00001d006084 | CGTCATCGGTAGGTCGTCAG | TCGATGTGCACGACTTGTCA |
| Zm00001d017153 | CTGTGCCTATGCGGGAGTAG | AAATCGGACGACTTGCTGGT |
| zma-miR408b-3p | GTAGGGGGCCATCAACAGAA | - |
| zma-miR408a | GACAGGGATGAGACAGAGCAT | - |
| zma-miR408b-5p | GTAGGGGGCCATCAACAGAA | - |
| zma-miR159c-3p | CTTCGATCCAATCCAGGAGGG | - |
| zma-miR528a-3p | CGAGGTGTGGCTGGAAGAAG | - |
| zma-miR166a-3p | AGGGGAATGTTGTCTGGCTCG | - |
| zma-miR528a-5p | CGAGGTGTGGCTGGAAGAAG | - |
| Nov-m0017-5p | TGAAGGGAGAAACACCGATGG | - |
| Nov-m0348-5p | ACAGCACTCGGCAAACAAGC | - |
| ZmSOD1 | TGAGGGTGTTACGGGGACTATCT | GTGAAGGTGGCAGTTCCATCATCTC |
| ZmCAT3 | GCGTTGAAACCTAACCCGAAAA | AAACCCTCCATGTGCCTGTAATCTT |
| ZmAPX2 | TTCATTCCACCCCCGTCTC | CCGTTACGCCTCAGCAAATCC |
| ZmPOD45 | TACCGACTTCGTCCCCATCT | GAGCTTGGCCATGTTCCTCT |
| ZmActin1 | ATGTTTCCTCCCATTGCCGAT | CCAGTTTCGTCATACTCTCCCTTG |
